# Dyslexie font does not benefit reading in children with or without dyslexia

**DOI:** 10.1007/s11881-017-0154-6

**Published:** 2017-12-04

**Authors:** Sanne M. Kuster, Marjolijn van Weerdenburg, Marjolein Gompel, Anna M. T. Bosman

**Affiliations:** 1 Behavioural Science Institute & Department of Special Education, Radboud University, Montessorilaan 3, 6525 HR Nijmegen, The Netherlands; 2 Braams & Partners, Center for Diagnostic Assessment and Treatment of Learning Disorders, Deventer, The Netherlands

**Keywords:** Dyslexia, Font, Reading

## Abstract

In two experiments, the claim was tested that the font “Dyslexie”, specifically designed for people with dyslexia, eases reading performance of children with (and without) dyslexia. Three questions were investigated. (1) Does the Dyslexie font lead to faster and/or more accurate reading? (2) Do children have a preference for the Dyslexie font? And, (3) is font preference related to reading performance? In Experiment 1, children with dyslexia (*n* = 170) did not read text written in Dyslexie font faster or more accurately than in Arial font. The majority preferred reading in Arial and preference was not related to reading performance. In Experiment 2, children with (*n* = 102) and without dyslexia (*n* = 45) read word lists in three different font types (Dyslexie, Arial, Times New Roman). Words written in Dyslexie font were not read faster or more accurately. Moreover, participants showed a preference for the fonts Arial and Times New Roman rather than Dyslexie, and again, preference was not related to reading performance. These experiments clearly justify the conclusion that the Dyslexie font neither benefits nor impedes the reading process of children with and without dyslexia.

Dyslexia is a problem with the acquisition of reading decoding and/or spelling. These problems are not accompanied by intellectual disabilities and remain despite intensive instruction and practice (Lyon, Shaywitz, & Shaywitz, 2003). Scientific research that aims at a causal explanation for reading and spelling problems (henceforth, dyslexia) is omnipresent (Wijnants, Hasselman, Cox, Bosman, & Van Orden, 2012). The strongest theoretical perspective on dyslexia is problems with phonology (Lyon et al., 2003; Shaywitz, Morris, & Shaywitz, 2008; Snowling, 2011; Vellutino & Fletcher, 2005; Vellutino, Fletcher, Snowling, & Scanlon, 2004). Phonology refers to the sound of spoken words. The ability to read requires the reader to decode each letter or letter cluster into its sounds or phonemes. Spelling requires the opposite, the segmentation of spoken words into its constituent phonemes and recoding them into visual symbols. Most remediation techniques, therefore, rely on strengthening phonologically based skills and proved to be successful when the approach includes visual presentation of written words (Duff & Clarke, 2011; Morais, 1991; Pennington, Peterson, & McGrath, 2009; Shaywitz et al., 2008; Snowling & Hulme, 2011; Tijms, 2011).

Although studies on the relationship between phonological skills and reading performance are relatively consistent, other research suggests a relationship between reading skill and vision (e.g., Irlen & Lass, 1989; Lovegrove, Martin, & Slaghuis, 1986; Orton, 1937; Stein & Walsh, 1997). In fact, the first written account on a child who appeared to be unable to read was by the British medical doctor Kerr (1897) who describes “…a boy with word blindness, who can spell the separate letters…” (p. 668). The term word blindness reveals the assumption of a visual origin of the reading and spelling problem.

The most persistent perspective on the relationship between a visual problem and reading performance is the magnocellular theory of dyslexia (Stein & Walsh, 1997). Visual sensitivity is insufficiently suppressed by the magnocellular system when reading, such that fixations from one place in the text to the other cannot be distinguished properly. Evidence for this assumption is equivocal (Amitay, Ben-Yehudah, Banai, & Ahissar, 2002; Stein, 2014; Stein & Walsh, 1997; see Gori & Facoetti, 2014, for an extensive overview of visual theories and potential, albeit debatable, treatments).

A somewhat older explanation put forward by Irlen and Lass (1989) is that poor readers may suffer from the so-called ‘scotopic sensitivity syndrome’, which refers to difficulties with a light source, glare, luminance, wave length, and black/white contrast. Irlen’s solution was to provide readers with tinted glasses or overlays. The evidence for the effectiveness of this treatment is also far from conclusive (e.g., Blaskey et al., 1990; Goldstand, Koslowe, & Parush, 2005; Henderson, Tsogka, & Snowling, 2013; Irlen & Lass, 1989).

Yet another reason for poor reading performance has been sought in typographical characteristics of text. The fact that children’s books for learning to read have a larger font size than books for more advanced readers reflects the idea that typographical properties affect the reading decoding process (Bernard, Chaparro, Mills, & Halcomb, 2002; Moret-Tatay & Perea, 2011; Wilkins, Cleave, Grayson, & Wilson, 2009; Woods, Davis, & Scharff, 2005). Legibility is thought to be affected by the following font characteristics: x-height, body size, character and interline spacing, and shape (i.e., weight, contrast, serif or sans serif types, italic, bold etc.). Each will be discussed in detail below.

## Typographical characteristics

The body size of a font refers to the height of the letter, measured from the lowest point of the descender until the top of the ascender, supplemented with a minimal amount of extra space which is needed in order to prevent that letters will touch other letters (Legge & Bigelow, 2011; Spelbrink, 2012). Font size is measured in points (pt). A point is about 0.353 mm or 1/72 of an inch. Fonts with body sizes between 9 and 12 points (Bolder et al., 1990) or between 9 and 11 points (Unger, 2006) are considered to be legible.

The x-height of a font is the height of the lowercase letter x, given a certain point size (Legge & Bigelow, 2011; Spelbrink, 2012). Fonts with a relatively large x-height are more legible than fonts with a smaller x-height (Bernard et al., 2002; Dobres, Chrysler, Wolfe, Chahine, & Reimer, 2017; Legge & Bigelow, 2011; Williams, 2001). Note, however, that extreme large or extreme small x-heights might have a negative impact on reading performances (Williams, 2001). In children’s books, x-height generally decreases with increasing reading age (Hughes & Wilkins, 2000).

The character and interline spacing of a font refer to the space between the letters and space between the lines and surrounding texts (Unger, 2006). Proportionally spaced fonts are considered to be more legible than monospaced (fixed character width; Gelderman, 1998). Gianotten (2014a), a Dutch expert in typography, maintains that a larger font, extra interline space, and adding extra space between letters positively contribute to legibility (see also, Perea, Panadero, Moret-Tatay, & Gómez, 2012). Hughes and Wilkins (2000) examined the combined effects of x-height, line spacing, word spacing, and line length. They found that reading speed of young children (aged 5 to 7 years) decreased when the size of the text decreased. However, reading speed of older children (aged 8 to 11 years) was not affected by the size of the texts. For accuracy, they found an effect for all age groups; more errors were made on smaller texts compared to larger (Hughes & Wilkins, 2000).

The shape of a font is determined by weight, contrast, being serif or sans serif, and italic or regular. Font weight refers to boldness. Font contrast is the ratio between thin and thick parts. Serif fonts are letters that have small lines added to the edge of letters, such as in Times New Roman or Cambria; fonts like Arial, Helvetica, and Calibiri are so-called sans serif fonts, because they lack lines trailing of the edges. Historically, serif fonts are considered to be more legible than sans serif fonts, since serifs make letters more distinguishable (McLean, 1980) and because it accentuates the end of the strokes (Rubinstein, 1988). Empirical findings concerning the effect on reading performances are, however, inconclusive (Gelderman, 1998; Unger, 2006). Some studies showed that sans serif fonts benefit reading outcomes (Kaspar, Wehlitz, von Knobelsdorff, Wulf, & von Saldern, 2015; Moret-Tatay & Perea, 2011; Woods et al., 2005). Others showed that serifs might only be beneficial for reading when the size of the letters is small, probably due to the fact that serif fonts have a slightly increased letters space (Arditi & Cho, 2005; Kaspar et al., 2015). Finally, we mention that italic or bold fonts may affect legibility negatively compared to regular fonts (Bolder et al., 1990; Williams, 2001).

Thus, legibility appears to be affected by a number of different characteristics of a font. These characteristics are, however, often interrelated. An example is a font that is changed from a regular one into an italic one. It is not just the angle of the letters that changes, but also the space between the letter; there is often less inter-letter space in an italic font than in a regular one. To study the effect of each of these properties separately is therefore not easy, and perhaps even impossible (Moret-Tatay & Perea, 2011; Russell-Minda et al., 2007).

Font characteristics and dyslexia

Only a few studies have focused on the effects of adjusting texts for people with dyslexia. A first conclusion that can be drawn from these studies is that font size matters. O’Brien, Mansfield, and Legge (2005) argue that children with dyslexia need a larger print size to support maximum reading speed compared to children with a typical reading development. A second conclusion is that no differences were observed in reading times of participants with dyslexia reading texts in sans serif or serif fonts (Rello & Baeza-Yates, 2013). We need to add that the mean age of the participants was 20 years with the youngest being 11 and the oldest 50. The effect of serif may turn out to be different for younger children with reading difficulties. The British Dyslexia Association (2014) recommends a sans serif font, although they do not refer to research to substantiate this advice. A third conclusion is that no differences in reading times were found for participants with dyslexia when texts were read in regular compared to italic fonts (Rello & Baeza-Yates, 2013). A final conclusion is that children with dyslexia may benefit from extra inter-letter spacing (Perea et al., 2012; Zorzi et al., 2012), but seem not to benefit from either monospaced or proportionally spaced fonts (Rello & Baeza-Yates, 2013). Note again that these null-findings in the work of Rello and Baeza-Yates (2013) are based on reading performance of older readers; typographical characteristics may have different effects in children who start to learn to read. Gianotten (2014b) suggested that dyslectics could benefit from larger fonts and more horizontal space and that larger interline space could lead to better reading performance. This assumption was recently challenged in a paper by van den Boer and Hakvoort (2015). They asked Dutch beginning (Grade 2) and more advanced readers (Grade 4), including a subsample of poor readers, to read a list of 144 words with varying interletter spacing. The results showed that none of the groups benefitted from extra, that is, above default interletter spacing of a 14-point Times New Roman font. Reduced interletter spacing (below the default value), however, affected reading fluency negatively in all groups.

Although the relationship between reading problems and font characteristics is not yet clear, recent developments provide an opportunity to study the role of typographical characteristics in more depth. A number of different fonts have been created specifically for readers with dyslexia, such as “OpenDyslexic” (Gonzalez, 2015), “ReadRegularTM” (French, 2003), “Sylexiad” (Hillier, 2008), and the “Dyslexie font” © of Boer (2015). The designers of these fonts emphasize two main aspects, namely, differences between letters and weighted bottoms. The focus in the experiments testing the effect of font concerned reading performance and font preference. Rello and Baeza-Yates (2013) compared reading performance of participants with dyslexia while using two fonts that were created for people with dyslexia (i.e., OpenDyslexic and OpenDyslexic Italic) and ten other fonts that were not specifically designed for people with dyslexia. Reading text in OpenDyslexic or in OpenDyslexic Italic did not lead to a decrease in reading time compared to the other ten fonts. Moreover, participants did not show a preference for the font OpenDyslexic. They preferred Verdana and Helvetica to OpenDyslexic (Rello & Baeza-Yates, 2013). Zikl et al. (2015) did not find significant differences either in reading speed and accuracy when the font OpenDyslexic was compared to Arial. Wery and Diliberto (2016) found similar results for reading rate and accuracy when they compared this font to Arial and Times New Roman.

The font that is the object of this study concerns Christian Boer’s Dyslexie font launched in 2009. Boer (2015) claims that Dyslexie-letter shapes are more distinguishable than other fonts as a result of adding extra weight to the bottom of the letters using more oblique letters, enlarged openings within letters, different shapes, enlarged ascenders and descenders, punctuation and capitals in bold, different heights for almost similar letters, increased x-height, and more space between the letters.

Although, this Dutch graphic designer claims that his font has positive effects on reading performance of dyslectics, there are no empirical studies that substantiate this. Boer (2011) supports his conjecture by referring to a study conducted by de Leeuw (2010), a master student from the University of Twente in the Netherlands. In her study, she had university students with (*n* = 21) and without dyslexia (*n* = 22) perform two reading tasks (i.e., word reading and pseudoword reading). They were asked to read both tests in Arial font as well as in Dyslexie font. Her conclusion was that no differences in reading speed between the task presented in Dyslexie font and Arial font emerged for either the students with or students without dyslexia. She also concluded that dyslectic students read more accurately in the Dyslexie font. Unfortunately, this could not be verified, because no proper statistical proof was presented. She also concluded that children with dyslexia had a more positive attitude towards the font Dyslexie. This conclusion could not be verified either, because again no statistical test was done to underpin this statement.

In 2013, another master study was conducted at the University of Twente concerning the effect of the Dyslexie font on reading performance. This study by Pijpker (2013) targeted reading skill of children with (*n* = 22) and without (*n* = 42) dyslexia aged between 8 and 12 years attending a regular primary school. Each child read four different texts, that each had two levels of difficulty. Again, reading in Dyslexie font did not lead to a decrease in reading time or reading errors compared to reading in Arial in either the readers with or without dyslexia. When they divided the dyslectic readers in a group that read relatively well (*n* = 9) and one that read relatively poorly (*n* = 13), they found an effect of font in the poor dyslectic readers group, that is, they made fewer errors when reading the Dyslexie font than when reading in Arial.

Recently, Marinus et al. (2016) compared Arial with Dyslexie in 39 English-reading low-performing readers at text level. In a condition where they had not controlled for spacing, the Dyslexie font seemed to have a slight advantage over Arial (7% more words were read per minute), but after the fonts were matched for within-word and between-word spacing the effect disappeared. The authors therefore concluded that if the font Dyslexie aids reading, it is not because of its shape but rather because of the increase in word spacing. Thus, the claim by the designer Boer that letter shapes are responsible for the assumed increased legibility is doubtful (Marinus et al., 2016).

Criticism on Boer’s claim also comes from the profession of typography. Gianotten (2014a, 2015), for example, questioned a couple of Boer’s typographical adaptations. One, the statement that the x-height of the Dyslexie font is larger than the x-height of other fonts is not correct. The x-height is a percentage of the capital height and body size (relative x-height). As shown in Table 1, the x-height of the Dyslexie font is 63% of the capital height and smaller than those of Arial and Times New Roman, and the x-height related to body size of the Dyslexie font is smaller than the relative x-height of the other two fonts.

**Table 1:** The absolute and relative sizes of letters in point size 12

	Font		
	Dyslexie	Times New Roman	Arial
Body size in mm.	5.6	4.2	4.2
Capital height in mm.	4.0	2.8	3.0
Lower case height in mm.	2.5	1.9	2.2
Lower case x-height related to body size in %	44.5	44.8	52.0
Lower case x-height related to capital height in %	63	67	73

Two, Bessemans (2012) and Gianotten (2014a, 2015) state that the body size of the Dyslexie font is larger than Boer (2015) claims. Extra interline space is added by the designer resulting in an amount of interline space that is not in accordance with point size. As a result of this, the notation of corps size is incorrect.

Three, according to Boer (2015), letters in the font Dyslexie are more distinct from each other than letters in other fonts and according to the designer this is its main effective characteristic. It has been suggested that specific subgroups, like children who are at the beginning of the reading process, people with visual disabilities, and children with dyslexia might benefit from fonts that are more distinctive (Pohlen, 2009; Unger, 2006). Williams (2001) argues that fonts that require more attention causes a text to be less readable, because the reader’s focus has to be shifted from the content of the text to the letter shapes. Wilkins et al. (2009) investigated this hypothesis and compared the reading performances in two fonts, namely Sassoon Primary and Verdana. Sassoon Primary letters are less distinctive than those of Verdana. Results showed that words written in Verdana font were read and searched more quickly by typical reading children than words written in Sassoon Primary font. Reading letters that are more distinctive might be beneficial to reading outcomes, this is in accordance with Boer’s statements. However, Marinus et al. (2016) have shown that, compared to the font Arial, the letters of the font Dyslexie are less distinct from one another.

Reading is an important skill, and problems in reading can affect a person’s life in several ways. If a change in font eases the problems of people who have difficulty reading or learning to read, then it is the duty of scientists to share this knowledge. It is, however, also a scientist’s duty to test claims made by people to avoid disappointments, investments, and false hope. This study aims to contribute to sound scientific knowledge regarding the assumed effects of the font Dyslexie.

## The present study

The main goal, therefore, is to investigate whether the Dyslexie font is, as claimed by Boer, beneficial for readers with dyslexia. In two experiments, we will compare the effect of the Dyslexie font with the sans serif font Arial, recommended by the British Dyslexia Association (2014), and a wide-spread used standard serif font Times New Roman. To increase the possibility of finding differences between fonts, participants will be asked to read lists of unconnected words as well as texts. Because the focus of this study was the effect of font shape rather than interline spacing and font size, we controlled for these typographical aspects in the best way possible (more details in the Method sections).

To avoid a confound with word or text characteristics, a full within-subjects design will be used. That is, participants will be asked to read the same texts or words written in different type faces. Experimental measures (see Method sections) will be taken to control for the so-called repetition effect, that is, the effect that a repeated reading of text or words is generally faster than a first reading (Lowder, Choi, & Gordon, 2013; Oliphant, 1983; Scarborough, Cortese, & Scarborough, 1977).

Given earlier empirical findings, we do not expect the Dyslexie font to be more helpful in easing reading than Arial and Times New Roman. It may, however, be the case that readers who prefer the Dyslexie font read more effectively in that font. We therefore asked all participants to indicate their preference for a particular font and subsequently related this preference to reading performance.

In sum, the main three questions of the present study are: (1) Does the Dyslexie font lead to faster and/or more accurate reading? (2) Do participants have a preference for the Dyslexie font? (3) Does a preference for a font affect reading performances? The group of participants in Experiment 1 consisted solely of children with dyslexia, whereas in Experiment 2 both children with and without dyslexia took part.

## Experiment 1

### Method

#### Participants

All participants were recruited from an assessment and treatment program at Braams & Partners, an institution for learning disorders in the Netherlands. They were diagnosed with dyslexia in this clinical center, according to the requirements of the Protocol for Dyslexia (Blomert, 2006). Children whose standardized test scores on word-reading tests were below the tenth percentile or participants with standardized test scores on word reading below the 16th percentile and whose standardized test scores on word-spelling tests were below the 10th percentile. Informed consent was obtained from all participants and their parents. A total of 170 Dutch children with dyslexia (100 boys and 70 girls) were included in this study. Ages ranged from 7;4 years to 12;4 years (*M* = 119.8 months, *SD* = 13.7). Participants were randomly assigned to two groups, which determined the sequence in which the examined fonts were presented. The age of participants in Order‘Arial - Dyslexie’ (*M* = 120.3 months, *SD* = 14.4) did not differ from that of participants in Order‘Dyslexie - Arial’ (*M* = 119.3 months, *SD* = 13.0), *t*(168) = .51, *p* = .61. Moreover, the average text-reading skills of the participants in the two groups were the same, *t*(168) = .32, *p* = .75. The text-reading skills, measured with the AVI reading cards (Krom, Jongen, Verhelst, Kamphuis, & Kleintjes, 2010) were comparable to those of an average pupil after 22 to 23 months of reading education (a little over 2 years).

##### Materials

Reading speed and accuracy at text level were measured using the test card AVI 3A (Visser, van Laarhoven, & ter Beek, 1994). This test card is part of the AVI test package [AVI toets pakket] that contains nine test cards of increasing order of difficulty. Scores were based on the time participants needed to read the text and the total number of errors they made. Reliability is evaluated as “acceptable” (Egberink, Janssen, & Vermeulen, 2014a). The test card AVI 3A contains short sentences with words of one to four syllables (Visser et al., 1994). Two versions of test card 3A were used: one printed in font Arial and one printed in font Dyslexie. To correct for extra interline spacing and larger letters in the font Dyslexie, adjustments were made to the interline spacing and body size. Therefore, we used Arial in body size 13 (capital height 3.28 mm, x-height 2.38 mm) with 1.35 vertical line spacing and the font Dyslexie in body size 12 (capital height 3.95 mm, x-height 2.51 mm) with 1.0 vertical line spacing.

##### Procedure

Both tests were administered in separate sessions. Half of the children were asked to read the card in font Arial first (Time 1) and then in font Dyslexie (Time 2), whereas the other half of the participants read the cards in the reversed order. The time interval between the two sessions was one to 2 weeks. Following the final reading at Time 2, the participants were shown both texts and asked whether they had a preference for one of the two fonts. To reduce bias, participants were not informed about the names of the fonts.

Results and Conclusion.

##### Reading performance

Two separate 2 (Order: Arial – Dyslexie versus Dyslexie – Arial) × 2 (Time: 1 versus 2) GLM repeated measures ANOVA’s with age as covariate was run on the scores of reading speed and reading accuracy, respectively. Time was a within-subjects factor and Order a between-subjects factor. The mean scores and standard deviations for reading speed and reading accuracy of the two Orders are presented in Table 2. As expected, the main effect of Time (i.e., repetition) on reading speed was highly significant, *F*(1, 167) = 33.86, *p* < .0001, *η* ^2^ = 17. Participants read the text at Time 2 considerably faster than at Time 1; the number of errors did not decrease significantly after repeated reading *F*(1, 167) = 1.08, *p* < .30, *η* ^2^ = .006. The effect of the covariate was also significant revealing that older students were faster readers *F*(1, 167) = 92.36, *p* < .0001, *η* ^2^ = .36, and made fewer errors, *F*(1, 167) = 34.55, *p* < .0001, *η* ^2^ = .17.

**Table 2:** Means (*M*) and standard deviations (*SD*) of the reading speed (in seconds) and reading errors at Times 1 and 2 for the two orders

		Time			
		1		2	
		*M*	*SD*	*M*	*SD*
Order*	*n*	Reading speed			
Arial – Dyslexie	85	95.1	51.6	78.5	34.0
Dyslexie – Arial	85	91.6	46.9	81.8	42.1
Total	170	93.4	49.2	80.2	38.2
		Reading Errors			
Arial – Dyslexie	85	4.1	3.2	3.6	3.2
Dyslexie – Arial	85	5.1	5.8	4.6	3.9
Total	170	4.6	4.7	4.1	3.6

*The Order Arial – Dyslexie refers to the fact that at Time 1 the participants read the text in font Arial and at Time 2, they read the text in font Dyslexie. For the Order Dyslexie – Arial the order was reversed.

The main effect of Order was not significant with respect to speed or accuracy, *F* < 1, and F(1,167) = 2.78, *p* = .10, *η* ^2^ = .02, respectively. Thus, the order in which the fonts were read did not affect reading speed and reading accuracy differentially. The interaction effect of Time and Order was also significant with respect to reading speed, *F*(1, 167) = 7.50, *p* < .007, *η* ^2^ = .04, but not with respect to accuracy, *F* < 1. This interaction will be investigated in the following analysis.

To investigate the question whether reading in the Dyslexie font aids reading separate GLM univariate analyses were conducted at Times 1 and 2 in which Font was a between-subjects factor and age in months served as covariate. Reading speed between Arial and Dyslexie did not differ significantly at Times 1 and 2, both *F*’s < 1. The analysis regarding accuracy yielded similar non-significant results, Time 1: *F*(1, 167) = 1.78, *p* = .18; Time 2: *F*(1, 167) = 3.04, *p* = .08.

#### Font preference

In the group of children that read Arial first and Dyslexie second, a preference for Arial was shown by 44.7% of the participants, 37.6% preferred the font Dyslexie, and the remaining 17.6% did not have a preference. In the group of children that read Dyslexie first and Arial second, 61.9% had a preference for Arial, 20.2% had a preference for Dyslexie, and 17.9% had no preference. The distribution of preferences was significantly different for the two groups, χ^2^ (2) = 6.76, *p* = .03). Therefore, the effect of preference on reading performance was tested separately for the two groups.

##### Reading performance and font preference

To test for the effect of preference on reading speed and reading errors eight separate Univariate GLM’s were conducted on reading speed and reading errors with font preference as independent variable. Age served again as covariate. Means, standard deviations, and *F* tests are presented in Table 3. The effect of the covariate was significant in all eight analyses. These effects have been discussed in the previous analyses.

**Table 3:** Means and standard errors (in brackets) of reading speed and reading error for the three preference groups at times 1 and 2

	Preference			
	Dyslexie	Arial	None	*F*-test
*n*	49	90	30	
Time 1: Reading speed				
Font Dyslexie	90.2 (8.4)	85.9 (4.8)	114.5 (9.0)	*F* (2, 80) = 3.95, *p* = .02*, *η* ^*2*^ = .09
Font Arial	89.6 (7.7)	92.3 (7.1)	114.0 (11.5)	*F* (2, 81) = 1.67, *p* = .20
Time 1: Reading errors				
Font Dyslexie	4.9 (1.4)	5.3 (0.8)	4.9 (1.5)	*F* < 1
Font Arial	3.5 (0.5)	4.2 (0.5)	5.0 (0.8)	*F* (2, 81) = 1.32, *p* = .27
Time 2: Reading speed				
Font Dyslexie	74.4 (5.1)	79.1 (4.7)	85.7 (7.5)	*F* < 1
Font Arial	87.8 (7.9)	76.8 (4.5)	94.4 (8.5)	*F* (2, 80) = 2.01, *p* = .14
Time 2: Reading errors				
Font Dyslexie	3.0 (0.5)	3.9 (0.5)	4.0 (0.8)	*F* < 1
Font Arial	5.2 (0.8)	4.6 (0.5)	4.3 (0.9)	*F* < 1

*The experimenter forgot to ask one child about her preference; analysis was therefore based on 169 children rather than 170.

Only one of the analyses reached significance, namely the one pertaining to Time 1, where students who read the font Dyslexie showed a significant main effect of preference. Subsequent Bonferroni corrected pairwise comparisons revealed no difference in reading speed of the text written in Dyslexie between those who preferred Dyslexie font and those who preferred Arial font, *p* = 1.0. Those without a preference, however, were slower in reading the text in Dyslexie than those who preferred Dyslexie, *p* < .02, but were not slower than the children who preferred Arial, *p* = .16.

##### Conclusion

Children with dyslexia read text written in font Dyslexie as fast as in font Arial and they made a comparable number of errors. Participants generally preferred Arial above Dyslexie and preference for either of the fonts was not related to better reading. Moreover, the second reading of the same text was indeed faster than the first time the text was read; the order in which the texts were read did not affect reading speed or reading accuracy.

## Experiment 2

In Experiments 2, the claim was tested that the font Dyslexie would benefit reading at the word level. The serif font Times New Roman was added, because of its widespread use in books. The same questions that were investigated in Experiment 1 were subject of investigation in Experiment 2. For reason explained below, the repetition effect in this experiment is probably limited or even absent.

### Method

#### Participants

The participant group in Experiment 2 consisted of children with dyslexia (*n* = 102) and without dyslexia (*n* = 45) who were all attending regular primary education. Like in Experiment 1, the participants with dyslexia were recruited from Braams & Partners, an institution for learning disorders. The selection criteria were equal to those in Experiment 1. Participants with dyslexia were recruited from Grades 2 to 6. Ages ranged from 7;9 years to 12;10 years (*M* = 123.9 months, *SD* = 14.1). Participants without dyslexia were recruited from Grades 2 to 4. Ages ranged from 7;6 years to 11;9 years (*M* = 110.2 months, *SD* = 12.9).

Informed consent was obtained from all participants and their parents. Participants were randomly assigned to one of three Font-order conditions (see Procedure). There were no significant differences between the age of the participants in the three Font-order conditions, *F* < 1. Moreover, the average text-reading skills of the participants in the three Font-order conditions were the same, *F* < 1. The text-reading skills, measured with the AVI reading cards (Krom et al., 2010) were comparable to those of an average pupil after 30 to 31 months of reading education.

##### Materials

To determine the word reading skills of the participants, the Three Minute Test [Drie Minuten Toets, DMT] (Krom et al., 2010) was used. This is a time-limited test consisting of three cards with words of different levels. The first card (i.e., Card 1) contains CV, VC, CVC words. The second card (i.e., Card 2) contains CCV, CCVC, CVCC, CCVCC, CCCVC, and CVCCC words. The third card (i.e., Card 3) contains multisyllabic words. The participants were asked to read aloud the words as fast and as accurately as possible. The number of words read correctly within 1 min determines the score. Reliability and validity are considered to be good (Egberink, Janssen, & Vermeulen, 2014b). There are three comparable versions available (i.e., versions A, B, and C). Each version contains the same words, but the words are presented in a different order. The fact that the versions are not identical may reduce or even nullify the repetition effect.

Although the versions are assumed to be comparable, all versions were converted in the three different fonts, to prevent inferences by the version of the test. In order to correct for the extra interline-spacing and larger letters in the font Dyslexie, adjustments were made to the interline spacing and body size. We tried to match for x-height. Therefore, for Cards 1 and 2, we used Arial point size 16 (capital height 4.04 mm, x-height 2.93 mm), Times New Roman in point size 16 (capital height 3.75 mm, x-height 2.53 mm), both with interline space 1.15, and Dyslexie in point size 13 (capital height 4.28 mm, x-height 2.72 mm) with interline space 0.85. For Card 3, we used Arial point size 14 (capital height 3.54 mm, x-height 2.57 mm), Times New Roman in point size 14 (capital height 3.28 mm, x-height 2.21 mm), both with vertical line spacing 1.30 and Dyslexie in point size 11 (capital height 3.62 mm, x-height 2.30 mm) with 1.0 vertical line spacing.

##### Procedure

Participants were tested at three different measurements (i.e., Times 1, 2, and 3) and read the cards in all three fonts. The time interval between sessions was one to 2 weeks. In Table 4 the design used in Experiment 2 is displayed. At Time 1, version A of the cards was applied, at Time 2 version B, and at Time 3 version C. After card reading at Time 3, participants were also asked whether they had a preference for one of the three fonts.

**Table 4:** Design of experiment 2

Condition	Time	Font	Card
ADT	1	Arial	1A, 2A, 3A
	2	Dyslexie	1B, 2B, 3B
	3	Times New Roman	1C, 2C, 3C
TAD	1	Times New Roman	1A, 2A, 3A
	2	Arial	1B, 2B, 3B
	3	Dyslexie	1C, 2C, 3C
DTA	1	Dyslexie	1A, 2A, 3A
	2	Times New Roman	1B, 2B, 3B
	3	Arial	1C, 2C, 3C

A, B, C indicates the version of Cards 1, 2, and 3.

### Results and Conclusion

#### Reading performance

A 2 (Group: children with dyslexia and children without dyslexia) by 3 (Font order: ADT, TAD, DTA) by 3 (Time: 1, 2, and 3) by 3 (Card: 1, 2, and 3) GLM repeated measures ANOVA with age as covariate was run on the number of words read correctly in 1 min on each card. Group and Font order were between-subjects factors, whereas Time and Card were within-subjects factors. Mean scores and results of GLM Univariate Analyses are presented in Table 5.

**Table 5:** Number of Participants per Group (n), Means (*M*) and Standard Deviations (*SD*) and Results of GLM Univariate Analyses at Times 1, 2, and 3 for the Effects of Font on Number of Words Read Correctly with Age as Covariate (N = 102)

	Font									
				Dyslexie	Arial		Times New Roman			
Group	Card	Time	*n*	*M (SD)*	*n*	*M (SD)*	*n*	*M (SD)*	*F-value*	*p-value*
Children with dyslexia	1	1	33	73.6 (23.0)	34	74.4 (19.2)	35	71.7 (20.1)	0.61	.55
		2	34	75.5 (20.8)	35	73.3 (20.9)	33	74.6 (24.6)	0.36	.70
		3	35	73.9 (20.0)	33	76.0 (24.5)	34	75.6 (19.7)	0.55	.58
	2	1	33	60.6 (25.3)	34	60.7 (18.0)	35	61.5 (22.9)	0.07	.94
		2	34	64.3 (21.3)	35	62.5 (22.5)	33	61.8 (25.6)	0.16	.86
		3	35	62.3 (22.3)	33	63.1 (26.5)	34	63.2 (20.2)	0.24	.79
	3	1	33	47.3 (21.6)	34	48.5 (16.4)	35	48.3 (19.9)	0.04	.96
		2	34	51.7 (18.7)	35	51.7 (22.4)	33	48.6 (22.6)	0.09	.92
		3	35	51.7 (21.3)	33	51.2 (23.0)	34	53.1 (19.3)	0.18	.84
Children without dyslexia	1	1	15	81.3 (20.2)	15	80.5 (15.3)	15	76.6 (22.0)	0.41	.66
		2	15	86.1(17.3)	15	81.9 (19.7)	15	86.2 (19.7)	0.43	.65
		3	15	80.3 (23.7)	15	82.7 (21.7)	15	83.1 (16.1)	0.14	.87
	2	1	15	70.8 (24.6)	15	70.3 (17.5)	15	63.3 (27.4)	0.70	.50
		2	15	70.8 (18.9)	15	65.3 (23.8)	15	72.8 (25.6)	0.60	.56
		3	15	68.1 (26.6)	15	74.3 (24.6)	15	68.2 (20.1)	0.27	.77
	3	1	15	56.8 (20.8)	15	55.5 (20.1)	15	49.0 (24.6)	0.85	.44
		2	15	56.7 (20.2)	15	51.8 (25.5)	15	59.9 (23.4)	0.58	.56
		3	15	52.5 (26.3)	15	60.9 (22.3)	15	57.6 (19.9)	0.62	.54

Apart from the significant effect of the covariate, *F*(1, 140) = 113.4, *p* < .0001, *η* ^2^ = .45, there was a significant main effect of Group, *F* (1, 140) = 44.2, *p* < .0001, *η* ^2^ = .24. Children without dyslexia had faster reading times than children with dyslexia on all three cards. The main effect of Card was also significant, *F*(2, 280) = 27.5, *p* < .0001, *η* ^2^ = .16. Post-hoc Bonferroni-corrected comparisons revealed that scores on Cards 1 were significantly higher than on Cards 2, which in turn were significantly higher than on Cards 3 (all *p*’s < .0001), which confirms the fact that orthographically complex words are more difficult to read than orthographically simple words (Card 1 is the easiest and 3 the most difficult). Neither the main effect of Font order nor the interaction between Font order and any of the other variables reached significant levels, indicating no differences with respect to ease of reading between the different fonts. To substantiate our finding, we present all Univariate GLM’s with age as covariate for each Card at each moment of time. Findings in Table 5 clearly show that fonts were equally easy (or difficult for that matter) to read.

#### Font preference

A preference for Arial was shown by 38.1% of the participants, 29.9% preferred Times New Roman, and 11.6% chose the font Dyslexie. The remaining 19.7% did not have a preference. A one-sample chi-squared test showed that fewer participants showed a preference for the font Dyslexie than expected, fewer participants showed no preference than expected, but more participants preferred Arial than expected and more participants preferred Times New Roman than expected (χ^2^ (3) = 23.92, *p* < .0001). The distribution of the preferences, however, was significantly different for children with and children without dyslexia (χ^2^ (3) = 10.54, *p* < .015). Of the children with dyslexia, 45.1% preferred Arial, 29.4% Times New Roman, and 10.8% Dyslexie. The remaining 13.7% had no preference. Of the children without dyslexia, 22.2% preferred Arial, 31.1% Times New Roman, and 13.3% Dyslexie. The remaining 33.3% had no preference.

#### Reading performance and font preference

To test for the effect of preference on reading performance, a 2 (Group: children with dyslexia vs. children without dyslexia) by 4 (Preference: Dyslexie vs. Arial vs. Times New Roman vs. None) × 3 (Font: Dyslexie vs. Arial vs. Times New Roman) GLM repeated measures ANOVA was conducted on the total number of words read correctly on the sum score of all three Cards. Font served as within-subjects variable, preference as a between-subjects variable, and age served as covariate. Apart from the significant effects of the covariate, *F*(1, 137) = 108.84, *p* < .0001, *η* ^2^ = .44, and the significant effect of Group, *F*(1, 137) = 28.10, *p* < .0001, *η* ^2^ = .17, none of the other effects reached significance: main effects of Font, *F*(2, 274 = 1.22, *p* = .30, *η* ^2^ = .009, and Preference, *F*(3, 137) = 1.15, *p* = .33, *η* ^2^ = .025. The non-significant interaction between Font and Preference, *F*(6, 274) = 1.10, *p* = .36, *η* ^2^ = .023, indicated no relationship between preference and number of words read correctly. Mean scores are presented in Table 6.

**Table 6:** Means and standard deviations (sd) of number of words read correctly for each of the four preference groups of children with and without dyslexia

	Children with dyslexia				Children without dyslexia			
Font		Dyslexie	Arial	Times New Roman		Dyslexie	Arial	Times New Roman
	*n*	*Mean (SD)*	*Mean (SD)*	*Mean (SD)*	*n*	*Mean (SD)*	*Mean (SD)*	*Mean (SD)*
Preference								
Dyslexie	11	192 (57)	183 (54)	193 (52)	6	168 (33)	173 (31)	160 (42)
Arial	46	180 (65)	183 (68)	180 (67)	10	199 (62)	206 (60)	204 (60)
Times New Roman	30	187 (63)	187 (63)	186 (64)	14	202 (49)	197 (49)	198 (53)
None	14	204 (63)	202 (58)	201 (62)	15	234 (79)	233 (76)	233 (77)

*The information on preference for one child with dyslexia was lost; analysis was therefore based on 146 children rather than 147

#### Conclusion

Despite the fact that the children without dyslexia were more than 1 year younger than children with dyslexia, they proved to be the better reader. This emphasizes the fact that the group of children who were diagnosed with dyslexia are indeed poor readers. Participants with and without dyslexia read as many words correctly during 1 min in the font Dyslexie as they did in Arial and Times New Roman. Neither of the groups showed a preference for the font Dyslexie; in fact more participants preferred Arial and Times New Roman above Dyslexie. Moreover, preference was not related to number of words read correctly. Finally, no effect of repeated reading occurred, which corroborates our assumption that the different order of words on the cards causes the cards to be similar but not identical.

## General discussion

This study was conducted to test whether the font Dyslexie could be beneficial for readers with dyslexia. Our first question concerned the effect of font on reading performances. Reading performance of children with and without dyslexia at word and text level are not better when they read in the font Dyslexie compared to the fonts Arial and Times New Roman. Although no effect on reading performance was found, it was possible that children with dyslexia displayed a preference for the font developed for children with dyslexia. Therefore, our second research question examined the preferences of children with and without dyslexia for a particular font. At text level, children with dyslexia preferred the font Arial over Dyslexie. The font Dyslexie was favored the least by children with and without dyslexia at the word level. The third research question was whether reading texts or words in the preferred font, would lead to better reading outcomes. We found no effect of reading a text or words in the preferred font on reading performances for dyslexic children or for children without dyslexia.

The results of both our experiments do not support the claim that the font Dyslexie facilitates the reading of dyslexic people. These findings are mostly in line with other Dutch studies conducted by de Leeuw (2010) and Pijpker (2013). First, de Leeuw (2010) found no effects of font on reading speed. However, these conclusions concerning reading speed were based on the effects of raw scores, that is, the number of words read correctly in 1 min. Normally the raw score is the number of words read in 1 min, irrespective of whether the words are read correctly or not. The “raw scores” in this study not only represent the reading speed, but also the accuracy of reading. Thus, in line with our results, de Leeuw found no effect of font on the number of words read correctly. Second, her conclusion that dyslectic students made fewer errors in the Dyslexie font version of the word-reading task (*M* = 1.3, *SD* = 1.4) than in the Arial font version (*M* = 1.7, *SD* = 1.3) could not be verified, because no proper statistical proof was presented. Finally, for pseudoword reading no effects were found either. Also in line with our findings are the results of Pijpker (2013), who found no effect at text level. The only effect in her study emerged when the group of dyslectic readers were divided into relatively good and poor readers. Note, that she did not make clear how this distinction was made. Thus, empirical evidence for Boer’s claim that the font Dyslexie has positive effects on the reading performances of people with dyslexia is lacking. His claim was based on just this one effect.

At this point, we need to discuss the fact that a major part of our standpoint is based on an abundance of null-findings, statistically a not very convincing argument. Note, however, that the experiments revealed three significant findings in line with previous research. First, in both experiments, a highly significant effect of age (the covariate) emerged; older children performed consistently better than younger children (e.g., Verhoeven & van Leeuwe, 2003). Second, Experiment 1 revealed a robust repetition effect as a result of repeated reading of identical materials, which converges with results from numerous studies on word and text reading (see for a meta-analysis, Thierren, 2004). Third, Experiment 2 revealed that reading single, isolated words in children with dyslexia was significantly worse than that of children without dyslexia. With respect to all null-findings related to the effect of font on reading performance, we would like to put forward that the majority of the statistical analyses revealed F-values smaller than 1, values that are not even approaching significance.

Both experiments showed that the font Dyslexie was not preferred by the children with dyslexia or by the children with normal reading development. These findings are in contradiction with the findings by de Leeuw (2010). She claimed that participants with dyslexia have a more positive attitude towards the font Dyslexie than typical reading students, based on the percentages of the answers given by the students with dyslexia and without dyslexia (i.e., on the four categories “unpleasant”, “neutral”, “pleasant”, and “very pleasant”) and reported no statistical analyses to underpin this conclusion. Furthermore, these differences in findings between the results of de Leeuw (2010) and the present study could be explained by a difference in procedure that has been followed. In de Leeuw’s research, participants were asked whether they preferred the font that was especially designed for people with dyslexia. In our research, the participants were not informed about the names of the fonts. The more positive attitude towards the font Dyslexie in de Leeuw’s research might be affected by her procedure. For people with dyslexia, the message that a simple solution like the use of a special font might lead to better reading outcomes can be rather hopeful. On top of that, de Leeuw (2010) presented only a selection of the results of the questionnaire measuring font preference in her thesis. In the appendix of her master thesis she reported all five questions asking the students how they experienced the font Dyslexie but again, no statistical analyses were given. When the students with dyslexia and the students with a normal reading development were asked whether they would use the font Dyslexie, no groups’ differences could be revealed (de Leeuw, 2010).

All in all, the font Dyslexie, developed to facilitate the reading of dyslexic people, does not have the desired effect. Children with dyslexia do not read better when text is printed in the font Dyslexie than when text is printed in Arial or Times New Roman. One of the claims of the designer of the font Dyslexie (Boer, 2015), was that it facilitates reading because of the difference between the letters. According to Boer (2015), letters in the font Dyslexie are more distinct from each other than letters in other fonts. Marinus et al. (2016), however, have shown that, compared to the font Arial, the letters of the font Dyslexie are less distinct from one another. The statement that the relative x-height of the font Dyslexie is larger compared to other fonts, is not correct either (Gianotten, 2014a, 2015). This might explain the finding that dyslexic children did not read faster or more accurate when reading in the font Dyslexie than in Arial.

Contrary to our findings, Marinus et al. (2016) did find that children with reading problems read English texts significantly faster in font Dyslexie than in Arial. However, they concluded that these results could not explained by the shape of the letters, but the broader spacing settings. In our experiments, we controlled for vertical spacing, but not for horizontal spacing. It would have been interesting if we had added this variable to examine the effects of spacing in words and texts on the reading performances on young children with dyslexia. Furthermore, we tried to control for the adaptations Boer made to the body size of the font by comparing the font Dyslexie with a smaller point size than the fonts Arial and Times New Roman; note that, these measurements do not exactly match. We do not believe that this has affected the results, because differences in x-height only emerge when the differences in x-heights are substantial (Bernard et al., 2002). As explained in the introduction, it is not possible to control for all variables at the same time.

In sum, the adaptations made to create the font Dyslexie have been disputed and the empirical findings also challenge the claim that Dyslexie is a better font for dyslectics. We haste to add that Dyslexie does not affect reading negatively. If children prefer to read texts in Dyslexie, there is no reason to discourage this. However, Wery and Diliberto (2016) warn for other negative effects using a font that claims to make reading easier. Readers who already have experienced major disappointments in learning to read may find it distressing when they fail to encounter positive effects of font characteristics. Also, purchasing the Dyslexie font and transferring materials into this font could also shift the focus (time and money) from interventions that have been proven effective (Wery & Diliberto, 2016). Therefore, we advise to focus on effective interventions for better reading outcomes. An effective intervention to improve reading in both accuracy and fluency contains (1) an expanded instruction in reading and (2) an increase of the number of words and text read (Bosman & Gijsel, 2007). To encourage reading, we believe that children should take advantage of the free available technical opportunities to adjust font type, font size and spacing options in digital texts to their personal preference. The purchase of a special font for dyslectics will then probably be unnecessary. Future research should focus on adjustments to texts that could be done easily by changing the settings in computers, laptops, tablets and e-readers to facilitate reading. A collaboration between the field of researchers in typography and psychology is recommended.
